# Role of Probiotics in Management of Depressive Symptoms and Cognitive Impairment in Patients With Depression: An Updated Analysis of Trials

**DOI:** 10.1002/brb3.71108

**Published:** 2025-11-29

**Authors:** Syed Ali Arsal, Aashish Kumar, Umer Iqbal, Shafin Bin Amin, Muhammad Aniq Amir, Muhammad Abrar Amir, Muhammad Ashir Shafique, Suhaib Ahmed, Laksh Kumar, Oluwatobiloba Israel Popoola, Inibehe Ime Okon

**Affiliations:** ^1^ Department of Medicine Shaheed Mohtarma Benazir Bhutto Medical College Karachi Sindh Pakistan; ^2^ Department of Medicine Dow Medical College Karachi Sindh Pakistan; ^3^ Department of Medicine Karachi Medical and Dental College Karachi Sindh Pakistan; ^4^ Department of Medicine Jinnah Sindh Medical University Karachi Sindh Pakistan; ^5^ Department of Medicine Shenyang Medical College Shenyang Liaoning China; ^6^ Department of Research Medical Research Circle (MedReC) Bukavu Democratic Republic of the Congo

**Keywords:** cognitive impairment, depression, meta‐analysis, probiotics, psychiatric illness

## Abstract

**Background and Objective:**

Depression is a global burden that causes mortality and morbidity in patients and affects millions of people worldwide. Conventional treatment of depression has limited efficacy in addressing cognitive symptoms. Probiotics, particularly psychobiotics, offer promise by modulating the gut–brain axis and improving both depressive symptoms and cognitive function. This meta‐analysis provides insights into the potential of probiotics in alleviating depression‐related symptoms and cognitive deficits in comparison with placebo.

**Method:**

A thorough literature search was performed using online resources such as PubMed, Embase, Cochrane, and Google Scholar. The seven randomized control trials (RCTs) met the inclusion criteria. The study was conducted in compliance with the PRISMA guidelines.

**Result:**

Patients receiving probiotics had significant alleviation of cognitive symptoms (*p* = 0.01; SMD, −0.90; 95% confidence interval [CI], −1.59 to −0.21; *I* ^2^ = 82%) in comparison to placebo. Likewise, the probiotic group had a significant reduction in depressive symptoms compared to the placebo group (*p* = 0.03; SMD, −0.55; 95% CI, −1.04 to −0.06; *I* ^2^ = 79%). Both results favored the probiotic group over the placebo group.

**Conclusion:**

In conclusion, research has demonstrated that probiotic supplementation can be a highly effective supplementary treatment for depression by effectively addressing psychological symptoms and cognitive problems. Its therapeutic potential in clinical settings should be explored by additional research to better understand its mechanisms.

## 1 Introduction

Psychiatric illness, especially depression, is becoming a global burden for causes of morbidity in patients, affecting 264 million individuals worldwide, and is known to be one of the major causes of disability in patients (Lam et al. 2014). The estimated lifetime risk of a severe depressive episode in the United States is currently close to 30% (Kessler et al. 2012), and it is estimated that 50% of suicidal events have background of mood disorders such as depression and bipolar disorder (Henriksson et al. 1993). Depressive illnesses can present with cognitive dysfunction, which may last long after the patient experiences symptom remission. Cognitive dysfunction can hinder and limit a patient's functioning in various domains and severely degrade its quality as they are enduring signs of depression (Czerwińska and Pawłowski 2020). In basic neuroscience studies, depression‐like behaviors and chronic stress have been linked to deficits in neuroplasticity, including synapse loss and neuronal atrophy in the hippocampus and medial prefrontal cortex (mPFC). As a multidomain neurobiological, cognitive, and psychological construct, neuroplasticity is relevant to depression and other disorders associated with negative affect, such as anxiety (Price and Duman 2020). Notably, a possible premorbid sign of depression is a decrease in verbal episodic memory. Ironically, in the context of contemporary therapeutic techniques, cognitive disorders are often ignored, despite their tremendous impact on daily functioning (Sousa et al. 2021).

The effectiveness of conventional major depressive disorder (MDD) treatments, which include psychotherapy and antidepressant drugs (7), in improving depression‐related cognitive impairment has been shown to be quite effective (Tiller 2013). However, there remains a need to develop novel and more effective treatment approaches that may simultaneously address both the emotional and cognitive aspects of depression at the same time. In this context, probiotics have emerged as viable intervention options (Liu et al. 2023). Studies have shown that probiotics can be a potential treatment for alleviating cognitive and depressive symptoms in patients with mild to severe forms of depression (Kim et al. 2021).

A peculiar group of probiotics known as psychobiotics impact immunological, humoral, neurological, and metabolic networks that regulate the gut–brain axis (GBA) and related behaviors and functions of the central nervous system (CNS) to enhance not just gastrointestinal (GI) function but also the ability to function as an antidepressant and anxiolytic (Jha et al. 2023).

Research on animals and rodents has demonstrated that the gut microbiota interacts with the host through immunological, neuroendocrine, and neurological pathways. These pathways are components of the brain–gut–microbiota axis, and preclinical studies have suggested that the microbiota uses this two‐way communication system to regulate behavior, brain development, and function (Kelly et al. 2016).

The objective of this meta‐analysis and systematic review was to thoroughly assess the impact of probiotic supplementation on cognition and associated brain functions in individuals with depression which was based on the evidence from the newer studies and provides the latest insight on this topic. We also aimed to evaluate the impact of probiotics on symptoms and indicators of depression in this population. This review offers a thorough and evidence‐based analysis of the possible advantages and restrictions of probiotic therapies for depression by synthesizing data from current clinical trials.

## 2 Methods

This review followed the PRISMA 2020 reporting guidelines (Page et al. 2021). Additionally, this review was registered on PROSPERO (CRD42024543034). No deviations from the registered protocol occurred.

### 2.1 Eligibility Criteria

This meta‐analysis examined only randomized control trials (RCTs) to evaluate the effect of probiotic supplementation on cognition and depressive symptoms in patients with depression. The inclusion criteria were as follows: (1) Participants with either a definite diagnosis of depression or any other psychiatric disorder with available data for the depression subgroup. (2) Studies should evaluate cognitive parameters such as memory, executive functionality, emotional process, and depression in patients. (3) A wide range of probiotics were identified in the search strategy, and all those affecting cognition and related brain functions were included. RCTs were excluded on the basis of the following criteria: (1) Observational studies, case reports, and all types of non‐randomized studies were excluded because of their potential for bias. (2) Studies conducted on species other than humans, and all in vitro studies were excluded. (3) Randomized trials that did not correspond to our PICOS framework were excluded.

### 2.2 Search Strategy and Data Sources

The search was conducted for randomized controlled trials published in 2023. Only studies published in English were included. A systematic protocol for the database search was developed to ensure a thorough review of the relevant literature. The search spanned databases such as PubMed, Google Scholar, and Embase. The search strategy was based on the use of Boolean operators (AND, OR) in conjunction with Medical Subject Headings keywords and free text terms. Table S1 shows the search string that was applied on PubMed. The goal of combining these terms was to find studies that focused on probiotics, depression, and cognition. First, we started with titles and abstracts of the compiled data to identify studies of potential relevance. A rigorous analysis of the full texts of the selected publications was then undertaken. This analysis was conducted independently by two researchers to determine their eligibility for inclusion in the meta‐analysis. In instances where disparities emerged, deliberations with a third researcher were conducted to achieve a consensus (Table S1).

### 2.3 Risk of Bias Assessment

**FIGURE 1:**
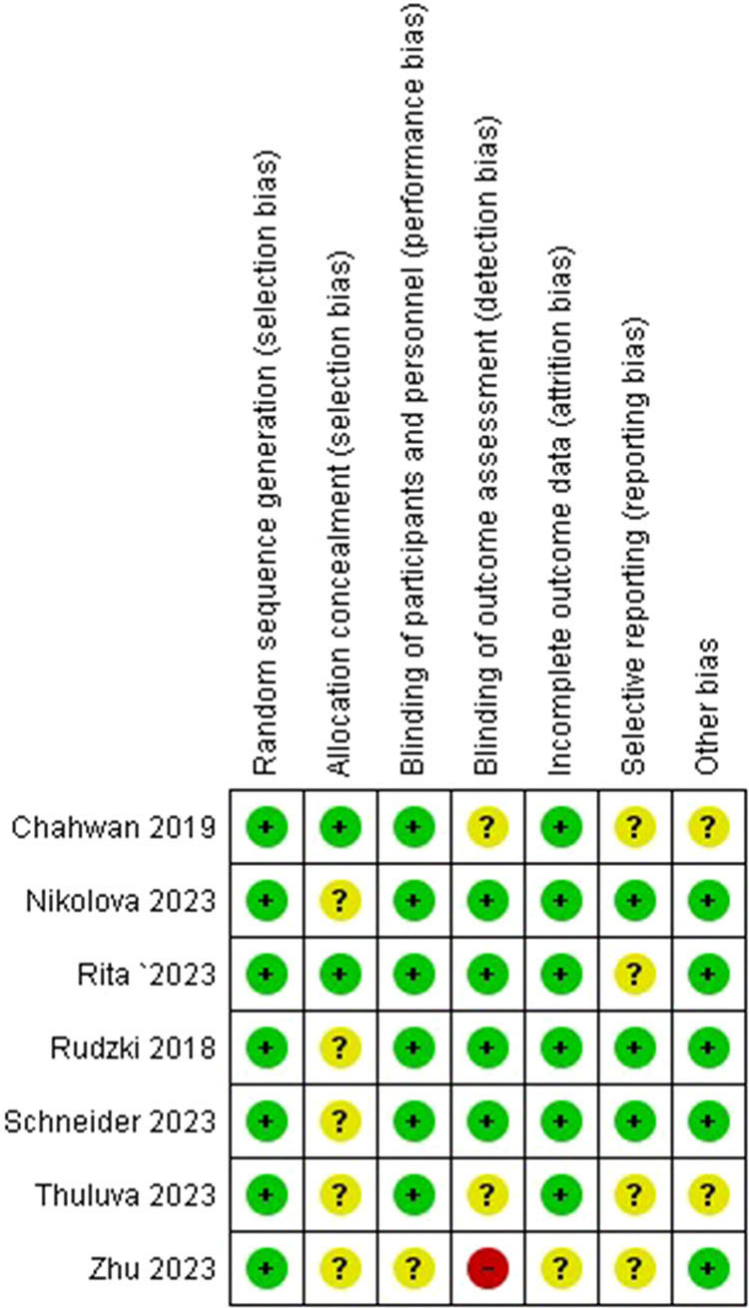
Risk of bias summary via traffic lights: review authors’ judgments about each risk of bias item for each included study.

Two selected researchers carefully evaluated the potential bias in randomized studies using the Cochrane Collaboration technique. This method was used to conduct an official evaluation of the quality of the chosen studies. The examination of bias included elements such as outcome blinding, accessibility of outcome data, exclusive reporting of outcomes in some circumstances, and the discovery of any other sources of bias. Studies were divided into three categories by evaluation: low risk of bias, high risk of bias, and unclear risk of bias because of incomplete data. When studies with a score higher than 1 were designated as “high risk,” a sensitivity analysis was also conducted to ensure the robustness of the findings. To address the possibility of publication bias, visual depiction using a funnel plot was employed. This comprehensive approach ensures meticulous evaluation of study quality and potential biases, leading to trustworthy and unbiased results (Figure 1).

### 2.4 Data Extraction

We gathered the baseline information, information related to intervention, patient number, and continuous data from the chosen RCT. Information on the evaluated cognitive parameters and the influence of probiotics on cognition was collected. This encompassed specifics of any noted alterations in cognitive performance, such as enhancements in memory, attention, and emotional processing. Data regarding the effects of probiotic supplementation on depression and depressive symptoms were compiled, including changes in depression scores and symptom severity. The overall conclusions or deductions made by each study regarding the correlation between probiotic supplementation, cognition, and depression were recorded.

### 2.5 Statistical Analysis

RevMan (Review Manager Version 5) was used to evaluate the data in this meta‐analysis. For every statistical analysis, a random‐effects model was used. The main aim of this study was to evaluate the effectiveness of probiotics in promoting favorable cognitive and behavioral changes related to depressive symptoms and signs in participants of the chosen clinical trials using quantitative methods. For cognitive and depressive symptom outcomes, we determined the mean difference (MD) and standard deviation (SD) values from each individual trial that was part of the review. These MD values were then input into RevMan 5. We computed the risk difference (RD) or relative risk (RR) using a 95% confidence interval (CI). We used the MD and 95% CI for continuous data. Statistical significance was set at *p* < 0.05. To visualize the data and evaluate statistical heterogeneity, we used a forest plot and *χ* ^2^ and *I* ^2^ tests (Higgins et al. 2003). If the *p* value was less than 0.05 or the *I* ^2^ exceeded 50%, significant heterogeneity was deemed to exist. When there was a lot of variation, we used a leave‐one‐out method to determine where it came from. This methodical approach guarantees the precision of our findings and fosters trust in their interpretation.

### 2.6 Publication Bias

To evaluate the possibility of publication bias in the included studies, we employed the Egger linear regression test. This statistical technique measures the asymmetry of the funnel plot based on the assumption that a lack of bias would result in a symmetrical funnel plot. A significant outcome suggests the presence of potential bias. It is important to note that the effectiveness of this test is limited when the number of studies is less than 10, so the results should be interpreted with care.

## 3 Results

### 3.1 Search and Selection Process

**FIGURE 2:**
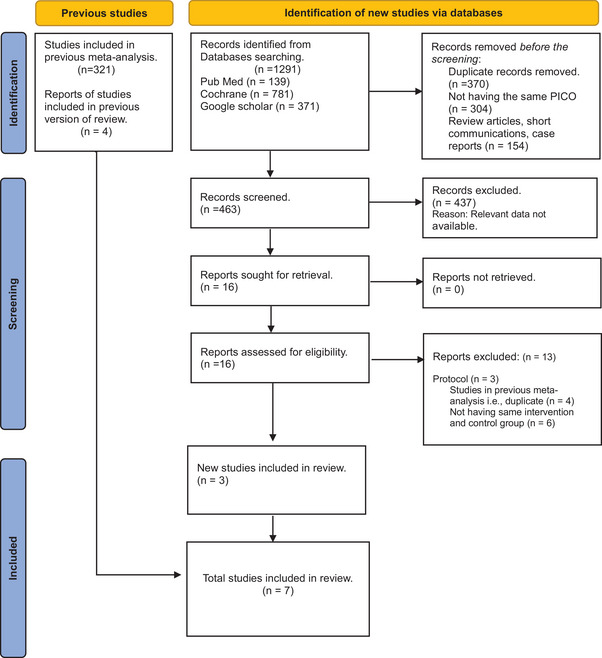
PRISMA flow chart of the included studies.

This process included a systematic approach to extract relevant studies for inclusion in our meta‐analysis. First, a detailed comprehensive search strategy was advanced involving keywords across multiple databases, including PubMed, Google Scholar, and the Cochrane Library. The initial search strategy gave 1291 results. Titles and abstracts of the recovered studies were screened to evaluate their acceptability according to predefined inclusion and exclusion criteria. After initial screening and removing 1275 irrelevant studies, full‐text articles of 16 conceivable related studies were obtained and further assessed for eligibility. Any disparities and differences in opinions were resolved through discussion and concurrence. Moreover, reference lists of the included studies were also searched manually to identify any significant studies missed during the initial search. Eventually, three reports fulfilling the inclusion criteria were selected, whereas those not meeting the criteria were excluded (13 trials). After merging the new RCTs with the parent meta‐analysis studies, we included a total of seven RCTs in our analysis. The entire search and selection process was supervised according to the PRISMA guidelines to certify pellucidity and transparency in study selection (Figure 2).

### 3.2 Quality Assessment

We placed a high priority on ensuring a thorough assessment of study quality in all our research initiatives. We have specifically used the Cochrane Table of Risk of Bias for RCTs, as the graphical and tabular presentations are provided in Table S2 and Figure S1.

### 3.3 Study Characteristics

The detailed characteristics of the studies included in the meta‐analysis can be reviewed in Table 1 which evaluates the effect of probiotics in persuading participants of productive changes in cognition and signs and symptoms of depression. It also encompasses details about the country, design, interventional group, control group, patients’ characteristics, and outcomes of the respective study. Our sample size included both males and females with a mean age between 22 and 39 years, covering an extensive range of age distribution. The total number of participants was 402, with a female‐to‐male ratio of 131:271 (0.48:1). This gender difference emphasizes the significance of sex‐related variance in the response of probiotic efficacy in reducing depression. Our study intervention included different multispecies probiotics for separate time periods in all studies, whereas the control group was placebo in all studies.

**TABLE 1:** Baseline characteristics of included studies.

Author	Year	Country	Design	Interventional group	Control group	Patients	Outcomes
Nikolova et al. (2023)	2023	London, the United Kingdom	RCT	Multistrain probiotic (8 billion colony‐forming units per day) containing 14 strains of *Bacillus subtilis*, *Bifidobacterium bifidum*, *Bifidobacterium breve*, *Bifidobacterium infantis*, *Bifidobacterium longum*, *Lactobacillus acidophilus*, *Lactobacillus delbrueckii* subsp. bulgaricus, *Lactobacillus casei*, *Lactobacillus plantarum*, *Lactobacillus rhamnosus*, *Lactobacillus helveticus*, *Lactobacillus salivarius*, *Lactococcus lactis*, and *Streptococcus thermophilus* (Bio‐Kult Advanced; ADM Protexin)	Placebo daily for 8 weeks	Adults aged 18–55 years with MDD taking antidepressant medication but having an incomplete response were studied	Probiotic group received greater improvements in depressive (HAMD‐17 scores) and anxiety (Hama scores) symptoms compared to placebo
Zhu et al. (2023)	2023	China	RCT	*Lactobacillus plantarum* JYLP‐326 twice per day for 3 weeks	Placebo (maltodextrin) twice per day for 3 weeks	Sixty anxious students were enrolled and randomly allocated to the placebo group and the probiotic group and thirty unanxious students with no treatments were assigned to a regular control group	*Lactobacillus plantarum* jylp‐326 reduced anxiety, depression, and insomnia symptoms in test‐anxious college students Placebo group showed higher gut microbiota, whereas jylp‐326 partly restored it Placebo group had increased *Bacteroides* and *Roseburia* but decreased *Prevotella* and *Bifidobacterium*, partially reversed by jylp‐326. Test anxiety correlated with altered fecal metabolites, which jylp‐326 assisted normalize. Changes in gut microbiota and fecal metabolites were related to anxiety symptoms, indicating a potential mechanism for the interventional group effectiveness
Gawlik‐Kotelnicka et al. (2023)	2023	Poland	RCT	Participants received a probiotic preparation containing *Lactobacillus helveticus* Rosell‐52 and *Bifidobacterium longum* Rosell‐175 over 60 days	Placebo consumed one daily	Sixty adult patients with depressive disorders were included in the study	The maintenance proportion within the probiotic vs. Placebo was 78.8% vs. 81.5%, with comparative dropout reasons. Adherence was full among all members, with no genuine side effects. For instance, minor antagonistic impacts like diarrhea were detailed more within the probiotic group. The statistical investigation included 44 members for psychometric scales, 46 for metabolic parameters, and 43 for inflammation markers. Baseline characteristics were the same and certifying study comparability. Metabolic and psychometric appraisals appeared potential change with probiotic mediation
Baião et al. (2023)	2022	The United Kingdom	RCT	Multispecies probiotics (*Bacillus subtilis*, *Bifidobacterium bifidum*, *Bifidobacterium breve*, *Bifidobacterium infantis*, *Bifidobacterium longum*, *Lactobacillus acidophilus*, *Lactobacillus delbrueckii* ssp. bulgaricus, *Lactobacillus casei* PXN, *Lactobacillus plantarum*, *Lactobacillus rhamnosus*, *Lactobacillus helveticus*, *Lactobacillus salivarius*, *Lactococcus lactis* ssp. lactis, *Streptococcus thermophilus* PXN) given for 4 weeks	Placebo	71 depressed patients were included	Probiotic intake improved emotional processing a little bit and reduced depression and affect specific cognitive changes positively
Chahwan et al. (2019)	2019	Australia	RCT	Probiotic group, were provided two sachets for each day of the trial, containing 2 g of freeze‐dried probiotic powder mixture (*Bifidobacterium bifidum*, *Bifidobacterium lactis*, *Lactobacillus acidophilus*, *Lactobacillus brevis*, *Lactobacillus casei*, *Lactobacillus salivarius*, *Lactococcus lactis*, and *Lactococcus*)	Placebo	71 patients with depressive symptoms were recruited and allocated sequentially over 12 months	Probiotic intake reduced cognitive irritability and improved depressive symptoms to some extent. Although it did not significantly change gut microbiota, it affects a psychological variable related to depression
Rudzki et al. (2019)	2019	Poland	RCT	SSRI with the probiotic *Lactobacillus plantarum* 299v (LP299v) (*n* = 40) for a period of 8 weeks	SSRI with the placebo of the probiotic (*n* = 39) for 8 weeks	79 patients with MDD were randomized and allocated to a double‐blind, placebo‐controlled trial	Improved attention and verbal memory (apt and cvlt) in lp299v group; decreased kynurenine (kyn) concentration; increased 3hkyn: kyn ratio; interaction for anthranilic acid (aa) concentration; no significant change in TNF‐α, IL‐6, IL‐1b, and cortisol concentrations. Improvement in cognitive functions; biochemical changes related to depression; no significant change in proinflammatory cytokines and cortisol. Overall, lp299v supplementation might have enhanced cognitive functions and modulated biochemical parameters related to depression without affecting proinflammatory markers
Schneider et al. (2023)	2023	Switzerland	RCT	A probiotic supplement (*Streptococcus thermophilus*, *Bifidobacterium breve* NCIMB 30441, *Bifidobacterium lactis*, *Bifidobacterium infantis*, *Lactobacillus acidophilus*, *Lactobacillus plantarum*, *Lacticaseibacillus. paracasei*, and *Lactobacillus delbrueckii* subsp and Thermophilus) was administered over 31 days in addition to treatment as usual for depression	Indistinguishable placebo containing maltose was administered over 31 days in addition to treatment as usual for depression	Sixty patients with MDD, of whom 43 entered modified intention‐to‐treat analysis	Improved immediate recall in verbal learning memory test (vlmt) in probiotic group; time × group interaction in hippocampus activation during working memory processing. Improvement in verbal episodic memory; remediated hippocampus function. Overall, probiotic supplementation with various strains enhanced verbal episodic memory and affected neural mechanisms underlying impaired cognition in MDD

Abbreviations: 3HKYN, 3‐hydroxykynurenine; AA, anthranilic acid; APT, attention performance test; CFU, colony‐forming units; CVLT, California verbal learning test; HAMA, Hamilton anxiety rating scale; HAMD‐17, 17‐item Hamilton Depression Rating Scale; IL‐1β, interleukin‐1 beta; IL‐6, interleukin‐6; KYN, kynurenine; MDD, major depressive disorder; RCT, randomized controlled trial; SSRI, selective serotonin reuptake inhibitor; TNF‐α, tumor necrosis factor‐alpha; VLMT, verbal learning memory test.

### 3.4 Outcomes

According to the defined PICO criteria, the outcomes of our study were the effect of probiotics on persuading productive changes in cognitive status and reduction in depressive signs and symptoms in the selected participants.

### 3.5 Effect of Probiotics and Productive Changes in Cognitive Status

**FIGURE 3:**
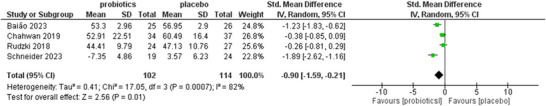
Forest plot of the effect of probiotics in improving cognitive function in the selected participants of the individual study.

During the meta‐analysis of our study, we analyzed four out of seven studies to assess the changes in cognitive status of individuals (Baião et al. 2023; Chahwan et al. 2019; Rudzki et al. 2019; Schneider et al. 2023). Our analysis results show that the study by Baião et al. delineated an MD of −1.23, with 95% CI of (−1.83, −0.62), favoring the probiotic group in improving cognitive function with a statistically significant CI. Chahwan et al. delineated an MD of −0.38, with a 95% CI of (−0.85, 0.09), favoring the probiotic group in improving cognitive function, but without statistical significance. Rudzki et al. delineated an MD of −0.26, with a 95% CI of (−0.81, 0.29), again favoring the probiotic group in improving cognitive function, but without statistical significance. Schneider et al. delineated an MD of −1.89, with 95% CI of (−2.62, −1.16) showing a significant improvement in cognitive functions in the probiotic group. The overall pooled effect of the four studies showed an MD of −0.90, with a 95% CI of (−1.59, −0.21), delineating the moderate improvement of cognitive function in participants assigned to the probiotic group. However, the most important result is that the overall pooled effect was statistically significant, as proved by the *Z* value of 2.56 (*p* = 0.01), explaining that the improvement in cognitive function in the probiotic group is of great significance. Additionally, a heterogeneity test was performed, which refers to the variation in study outcomes between studies (Chi^2^ = 17.05, degrees of freedom [df] = 3, *p* = 0.0007), indicating high heterogeneity (*I* ^2^ = 82%). However, for high heterogeneity, a sensitivity analysis was performed to assess the risk of bias using the leave‐one‐out method. Figure 3 demonstrates the forest plot that explains the MD and its 95% CI regarding the effect of probiotics on cognitive function.

### 3.6 Effect of Probiotics and Reduction in Depressive Signs and Symptoms

**FIGURE 4:**
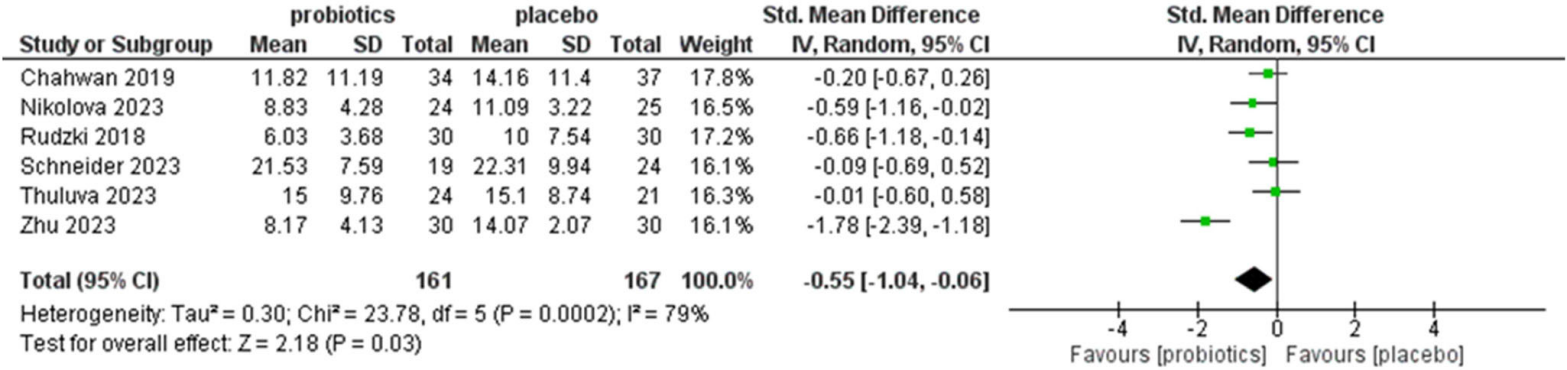
Forest plot of the effect of probiotics in reducing depressive signs and symptoms in the selected participants of the individual study.

Six studies were included in the analysis, which predicts reduction in depressive signs and symptoms in probiotic group compared to placebo (Chahwan et al. 2019; Gawlik‐Kotelnicka et al. 2023; Nikolova et al. 2023; Rudzki et al. 2019; Schneider et al. 2023; Zhu et al. 2023). Interpreting the data obtained from six studies, the study by Chahwan et al. (2019) indicated an MD of −0.20, with a 95% CI of (−0.67, 0.26), suggesting a reduction in depressive signs and symptoms in the probiotic group, but with a statistically insignificant CI. Nikolova et al. (2023) reported standard MD of −0.59 with 95% CI of (−1.16, −0.02), advocating reduction in depressive signs and symptoms in probiotic group with statistically significant CI. Rudzki et al. (2019) denoted a standard MD of −0.66, with a 95% CI of (−1.18, −0.14), recommending a minor decrease in depressive signs and symptoms in the probiotic group with statistical significance. Schneider et al. (2023) demonstrated standard MD of −0.09 with 95% CI of (−0.69, 0.52), suggesting insignificant decrease in reducing depressive signs and symptoms comparing both groups. Thuluva et al. (Gawlik‐Kotelnicka et al. 2023) delineated standard MD of −0.01 with 95% CI of (−0.60, 0.58), explaining negligible decrease in depressive symptoms with statistically insignificance. Zhu et al. (2023) displayed standard MD of −1.78 with 95% CI of (−2.38, −1.18), favoring reduction in depressive signs and symptoms in probiotic group with statistically significant CI. The overall effect of pooled analysis is the standard MD of −0.55, with a 95% CI of (−1.04, −0.06), clearly favoring the probiotic group in reducing depressive signs and symptoms in the included participants. More importantly, the overall effect is statistically significant, as manifested by the *Z* value of 2.18 (*p* = 0.03), showing that the reduction in depressive signs and symptoms are statistically significant. Moreover, heterogeneity test was also performed, which detects the variation among studies, demonstrating a chi‐squared value of 23.78 with 5 df and *p* value of 0.0002, showing high heterogeneity (*I* ^2^ = 79%), indicating statistically significant results but inconsistency between individual studies. Due to high heterogeneity, sensitivity analysis was performed to rule out any risk of bias using the leave‐one‐out method, which significantly drops to low heterogeneity. Figure 4 presents the forest plot, which illustrates the MD and its 95% CI regarding the effect of probiotics on depressive signs and symptoms.

## 4 Leave One Out of the Analysis and Publication Bias

### 4.1 Effect of Probiotics and Productive Changes in Cognitive Status

**FIGURE 5:**
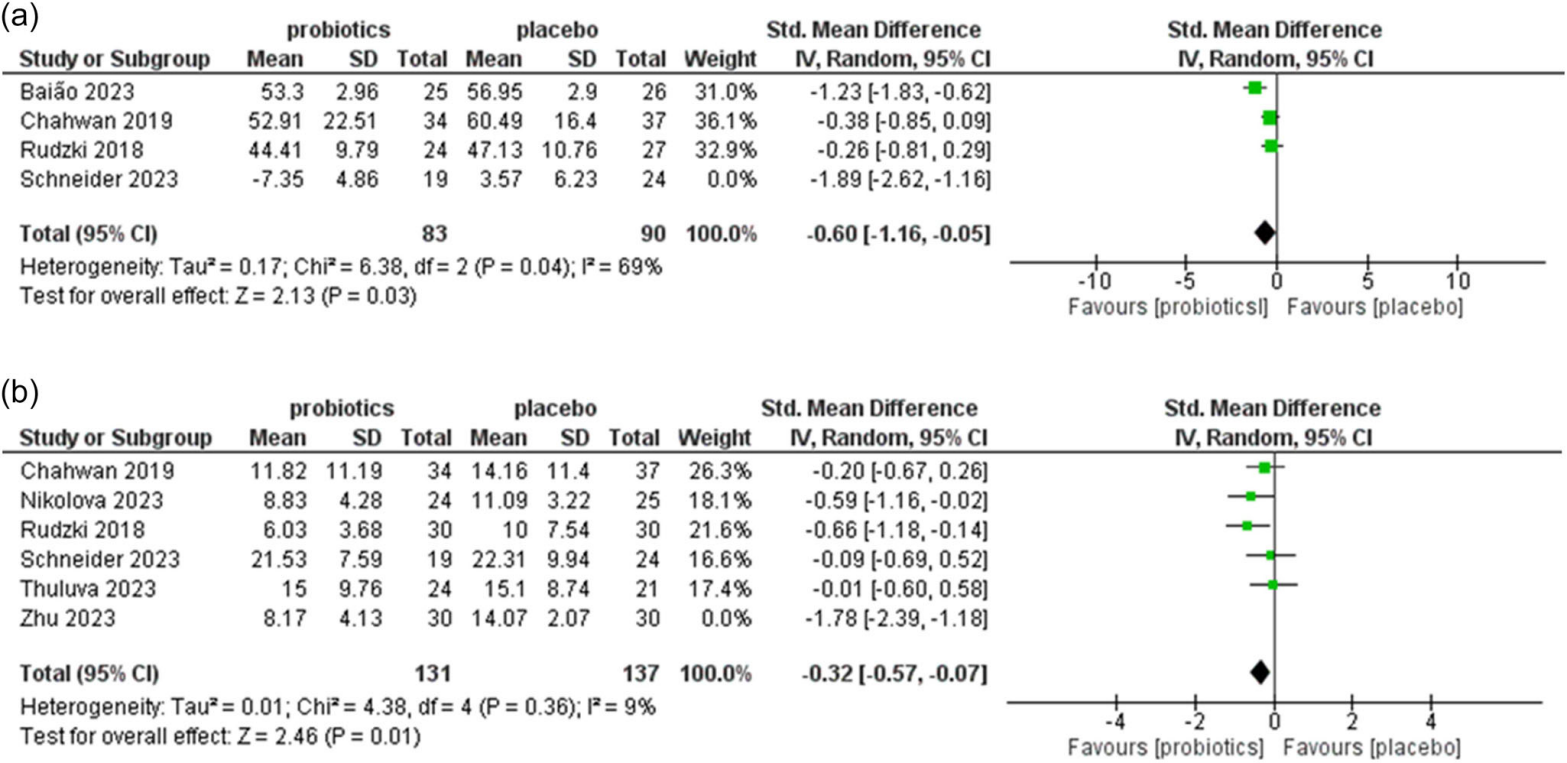
(a) Leave one out the analysis of the effect of probiotics in improving cognitive functions in the selected participants of the individual study. (b) Leave one out the analysis of the effect of probiotics in reducing depressive signs and symptoms in the selected participants of the individual study.

The pooled analysis of four studies showed a standard MD of −0.60 (95% CI −1.16, −0.05; Chi^2^ = 6.38, df = 2, *p* = 0.04; *I* ^2^ = 69%). As the heterogeneity was high, a leave‐one‐out analysis was performed, which lowered the heterogeneity from high to moderate with significant results. Schneider et al. (2023) was ruled out as the cause of high heterogeneity. Nevertheless, a small sample size and small number of studies can hinder the validity of the results. Figure 5a illustrates the forest plot of the leave‐one‐out analysis. Funnel plot for effect of probiotics and productive changes in cognitive status as visual depiction of the bias (see Figure S2).

### 4.2 Effect of Probiotics and Reduction in Depressive Signs and Symptoms

The pooled analysis of six studies revealed a standard MD of −0.32, with a 95% CI of (−0.57, −0.07), indicating results of the heterogeneity test as a chi‐squared value of 4.38, with 4 df, *p* value of 0.36, specifying low heterogeneity *I* ^2^ = 9%. As described above, heterogeneity was high, which significantly dropped to low. Zhu et al. (2023) was ruled out as the cause of heterogeneity. However, the small size and number of studies can disrupt the certainty of the results. Figure 5b shows the forest plot of the leave‐one‐out analysis. Funnel plot for effect of probiotics and reduction in depressive signs and symptoms, as visual depiction of the bias (See Figure S3).

## 5 Discussion

Our meta‐analysis is an enhancement of the study titled “Effect of probiotic supplementation on cognition and depressive symptoms in patients with depression” (He et al. 2023) that was published in 2023. The primary objective of our analysis was to delve deeper into the impact of probiotics on depression and various cognitive aspects, such as memory, executive functionality, and emotional processing.

In our study (Nikolova et al. 2023; Zhu et al. 2023), we observed a marked improvement in the signs and symptoms of depression in the intervention group. Similarly, probiotics also appeared to aid cognitive functions, such as face recognition (Rudzki et al. 2019; Schneider et al. 2023), in the intervention group.

Several theories explain how probiotics could potentially alleviate depressive symptoms. One of the key hypotheses is that probiotics aid in reducing inflammation, which in turn brings about positive changes in the gut–brain connection. This helps maintain emotional well‐being and alleviate depressive symptoms (Schneider et al. 2023). Some studies have shown that changes in the gut microbiota brought about by probiotics are helpful in alleviating depression. Antibiotics, which also affect the gut microbiota, have been observed to exacerbate depression in some patients, supporting the relationship between gut biota, neurotransmitters, and ultimately, depression (Xu et al. 2023). Cognition, the second outcome addressed in our meta‐analysis, was also found to be positively affected in patients on probiotics versus those in the control group (Xiang et al. 2022). It has been found that probiotics, which bring about changes in the gut microbiota, decrease the concentration of kynurenine in plasma, thereby helping improve cognitive functions (Handajani et al. 2023; Rudzki et al. 2019). The impact of probiotics on the intestinal barrier and permeability, along with a decrease in intestinal translocation (Zareie et al. 2006), modulation of indoleamine activity, and reduction in inflammation, all contribute to a decrease in kynurenine concentration (Ağagündüz et al. 2023; Rudzki et al. 2019).

Furthermore, the hippocampus, which is a crucial structure for depression‐related cognitive decline, is strongly associated with gut microbiota. This association influences hippocampus‐dependent learning, behavior, and memory, making it a significant point of discussion when establishing a relationship between cognitive function and probiotics (Salami and Soheili 2022). Probiotics are said to enhance learning and memory by raising butyrate, which raises BDNF and lowers the levels of proinflammatory cytokines in the hippocampal regions (Romo‐Araiza et al. 2018).

Our meta‐analysis builds on previous research and provides further evidence supporting the potential benefits of probiotics in managing depression and improving cognitive function. However, further research is needed to fully understand the mechanisms involved and determine the most effective types and doses of probiotics for these purposes.

In essence, our meta‐analysis contributes to a growing body of evidence that favors the use of probiotics in clinical practice when dealing with depression and cognitive function. If further research is carried out, it can be helpful, especially in treating patients suffering from mild and moderate intensities of depression and having new onset of symptoms. Patients suffering from depression have postdepression cognitive decline that includes decreases in concentration, learning, memory, and executive functionality that add to the misery they are going through (Roberts et al. 2022). Using probiotics has been shown to be advantageous for these patients and can potentially help them recover swiftly. We think that adding probiotics to treat these two conditions can open new doors to look into the GBA and potentially assist us to understand in more detail the cause of disease and give us a new treatment approach to encounter them. This also allows us to look into the depression symptoms people confront in everyday life, and how just by bringing up a little modification in diet can aid us in raising our mood. However, further research is needed to discover what type of microbiota brings these changes, the dosage and nature of probiotics to be used, and how to monitor their effect when used in the general population.

The efficacy of probiotics has been a significant topic of interest in recent years, with a wealth of research underscoring their potential benefits in the treatment of depression. Studies have consistently shown that probiotics are more effective than prebiotics, leading to notable improvements in depressive symptoms (Liu et al. 2019). Age appears to play a crucial role in the efficacy of probiotics, with positive results observed in individuals under 60 years of age, whereas those over 65 years of age show no significant benefits (Tsai et al. 2023).

Our study echoes these findings, focusing on patients younger than 60 years and highlighting the pronounced effects of probiotics on this age group. It is important to note, however, that some studies have not observed changes in depression severity, which may be attributed to the variety of depression scales used. Our research, utilizing the Depression Anxiety Stress Scales (DASS) and Hamilton Rating Scale, found significant improvements, suggesting that the choice of scale can influence the outcomes. In terms of cognitive function, studies involving patients with Alzheimer's disease and minimal cognitive impairment have shown promising results, aligning with our analysis (Den et al. 2020). However, there are also studies that report no cognitive improvements following probiotic, prebiotic, or fermented food interventions (Marx et al. 2020). This finding indicates the need for more nuanced research to understand the full impact of these interventions on mental health.

In our comprehensive study, we encountered a notable degree of heterogeneity, which we have determined is largely due to the diverse formulations, strains, and dosages of probiotics utilized across different trials. This variability is a critical factor in the interpretation of results. Moreover, inconsistency in the measurement scales, such as the Hamilton Rating Scale of Depression in most studies (Nikolova et al. 2023; Zhu et al. 2023), is used for depression assessment, whereas the DASS scaling system is used with the Beck Depression Index (BDI‐II) and Beck Anxiety Inventory (BAI) (Chahwan et al. 2019) to report outcomes across the studies further compounds this heterogeneity, making it challenging to draw uniform conclusions.

To address this issue methodically, we implemented a “leave‐one‐out” analytical approach. This technique involves sequentially removing one study at a time to assess the impact on overall heterogeneity. Notably, when we excluded the study conducted by Schneider in 2023, we observed a significant reduction in heterogeneity. This suggests that the particular methodology or population used in Schneider's study may differ substantially from the others. Similarly, the removal of Zhu's 2023 study from the analysis also resulted in a marked decrease in heterogeneity levels, especially when examining depressive signs and symptoms within the intervention group. Additionally, the studies involved people from different races and regions and did not consider the comorbidities that patients have and can potentially affect the results.

These findings underscore the complexity of synthesizing data from multiple sources and highlight the importance of standardization methodologies in future research. Refining our approach to account for these variations can enhance the reliability of our meta‐analysis and provide clearer guidance for clinical practice. Because earlier studies were not re‐searched (as they were included in the parent meta‐analysis), some relevant older data may not have been reassessed. Most study participants were male (female‐to‐male ratio 0.48:1), limiting generalizability. Probiotic effects may vary by sex; therefore, the findings must be interpreted with caution. Although subgroup/meta‐regression was considered, the limited number of included studies prevented formal analysis. Through such meticulous scrutiny and adaptation, we can pave the way for more targeted and effective interventions for the treatment of depression and its cognitive manifestations.

## 6 Conclusion

In conclusion, research has demonstrated that probiotic supplementation can be a highly effective supplementary treatment for depression by effectively addressing psychological symptoms and cognitive problems. Its therapeutic potential in clinical settings should be explored by additional research to better understand its mechanisms.

## Author Contributions

Screening was performed by Shafin Bin Amin and Laksh Kumar. Extraction and analysis were done by Syed Ali Arsal and Aashish Kumar. Muhammad Aniq Amir, Muhammad Abrar Amir, Umer Iqbal, and Suhaib Ahmed wrote manuscript. Editing and compilation were done by Muhammad Ashir Shafique and Oluwatobiloba Israel Popoola. Conceptualization was done by Syed Ali Arsal during the whole process. Supervision and critical thinking were done by Inibehe Ime Okon.

## Funding

The authors have nothing to report.

## Ethics Statement

The authors have nothing to report.

## Consent

The authors have nothing to report.

## Conflicts of Interest

The authors declare no conflicts of interest.

## Transparency Statement

The lead author, Inibehe Ime Okon, affirms that this manuscript is an honest, accurate, and transparent account of the study being reported; that no important aspects have been omitted; and that any discrepancies from a study as planned (and, if relevant, registered) have been explained.

## Supporting information




**Figure S1**. Risk of bias graph.
**Figure S2**. Funnel plot of effect of probiotics in improving cognitive functions in the selected participants of individual study.
**Figure S3**. Funnel plot of effect of probiotics in reducing depressive signs and symptoms in the selected participants of individual study.

**Table S1**. Search strategies of all databases.
**Table S2**. Risk of bias assessment by Cochrane risk of bias tool.

## Data Availability

The data that support the findings of this study are available from the corresponding author upon reasonable request.

## References

[brb371108-bib-0001] Ağagündüz, D. , E. Çelik , Ö. Cemali , et al. 2023. “Probiotics, Live Biotherapeutic Products (LBPs), and Gut–Brain Axis Related Psychological Conditions: Implications for Research and Dietetics.” Probiotics and Antimicrobial Proteins 15: 1014–1031. 37222849 10.1007/s12602-023-10092-4

[brb371108-bib-0002] Baião, R. , L. P. Capitão , C. Higgins , M. Browning , C. J. Harmer , and P. W. J. Burnet . 2023. “Multispecies Probiotic Administration Reduces Emotional Salience and Improves Mood in Subjects With Moderate Depression: A Randomised, Double‐Blind, Placebo‐Controlled Study.” Psychological Medicine 53: 3437–3447. 35129111 10.1017/S003329172100550X PMC10277723

[brb371108-bib-0003] Chahwan, B. , S. Kwan , A. Isik , S. van Hemert , C. Burke , and L. Roberts . 2019. “Gut Feelings: A Randomised, Triple‐Blind, Placebo‐Controlled Trial of Probiotics for Depressive Symptoms.” Journal of Affective Disorders 253: 317–326. 31078831 10.1016/j.jad.2019.04.097

[brb371108-bib-0004] Czerwińska, A. , and T. Pawłowski . 2020. “Cognitive Dysfunctions in Depression—Significance, Description and Treatment Prospects.” Psychiatria Polska 54: 453–466. 33038880 10.12740/PP/OnlineFirst/105415

[brb371108-bib-0005] Den, H. , X. Dong , M. Chen , and Z. Zou . 2020. “Efficacy of Probiotics on Cognition, and Biomarkers of Inflammation and Oxidative Stress in Adults With Alzheimer's Disease or Mild Cognitive Impairment—A Meta‐Analysis of Randomized Controlled Trials.” Aging (Albany NY) 12: 4010–4039. 32062613 10.18632/aging.102810 PMC7066922

[brb371108-bib-0006] Gawlik‐Kotelnicka, O. , A. Margulska , A. Skowrońska , and D. Strzelecki . 2023. “PRO‐DEMET Randomized Controlled Trial on Probiotics in Depression‐Pilot Study Results.” Nutrients 15: 1400. 36986132 10.3390/nu15061400 PMC10058314

[brb371108-bib-0007] Handajani, Y. S. , A. Hengky , E. Schröder‐Butterfill , E. Hogervorst , and Y. Turana . 2023. “Probiotic Supplementation Improved Cognitive Function in Cognitively Impaired and Healthy Older Adults: A Systematic Review of Recent Trials.” Neurological Sciences 44: 1163–1169. 36529793 10.1007/s10072-022-06540-8

[brb371108-bib-0008] He, J. , L. Chang , L. Zhang , W. Wu , and D. Zhuo . 2023. “Effect of Probiotic Supplementation on Cognition and Depressive Symptoms in Patients With Depression: A Systematic Review and Meta‐Analysis.” Medicine 102: e36005. 38013351 10.1097/MD.0000000000036005 PMC10681621

[brb371108-bib-0009] Henriksson, M. M. , H. M. Aro , M. J. Marttunen , et al. 1993. “Mental Disorders and Comorbidity in Suicide.” American Journal of Psychiatry 150: 935–940. 8494072 10.1176/ajp.150.6.935

[brb371108-bib-0010] Higgins, J. P. , S. G. Thompson , J. J. Deeks , and D. G. Altman . 2003. “Measuring Inconsistency in Meta‐Analyses.” BMJ 327: 557–560. 12958120 10.1136/bmj.327.7414.557 PMC192859

[brb371108-bib-0011] Jha, P. , N. Dangi , and S. Sharma . 2023. “Probiotics Show Promise as a Novel Natural Treatment for Neurological Disorders.” Current Pharmaceutical Biotechnology 25: 799–806. 10.2174/0113892010261604230919170143 37877144

[brb371108-bib-0012] Kelly, J. R. , Y. Borre , C. OB , et al. 2016. “Transferring the Blues: Depression‐Associated Gut Microbiota Induces Neurobehavioural Changes in the Rat.” Journal of Psychiatric Research 82: 109–118. 27491067 10.1016/j.jpsychires.2016.07.019

[brb371108-bib-0013] Kessler, R. C. , M. Petukhova , N. A. Sampson , A. M. Zaslavsky , and H. U. Wittchen . 2012. “Twelve‐Month and Lifetime Prevalence and Lifetime Morbid Risk of Anxiety and Mood Disorders in the United States.” International Journal of Methods in Psychiatric Research 21: 169–184. 22865617 10.1002/mpr.1359 PMC4005415

[brb371108-bib-0014] Kim, C. S. , L. Cha , M. Sim , et al. 2021. “Probiotic Supplementation Improves Cognitive Function and Mood With Changes in Gut Microbiota in Community‐Dwelling Older Adults: A Randomized, Double‐Blind, Placebo‐Controlled, Multicenter Trial.” Journals of Gerontology Series A, Biological Sciences and Medical Sciences 76: 32–40. 32300799 10.1093/gerona/glaa090 PMC7861012

[brb371108-bib-0015] Lam, R. W. , S. H. Kennedy , R. S. McLntyre , and A. Khullar . 2014. “Cognitive Dysfunction in Major Depressive Disorder: Effects on Psychosocial Functioning and Implications for Treatment.” Canadian Journal of Psychiatry Revue Canadienne De Psychiatrie 59: 649–654. 25702365 10.1177/070674371405901206 PMC4304584

[brb371108-bib-0016] Liu, L. , H. Wang , X. Chen , Y. Zhang , H. Zhang , and P. Xie . 2023. “Gut Microbiota and Its Metabolites in Depression: From Pathogenesis to Treatment.” EBioMedicine 90: 104527. 36963238 10.1016/j.ebiom.2023.104527 PMC10051028

[brb371108-bib-0017] Liu, R. T. , R. F. L. Walsh , and A. E. Sheehan . 2019. “Prebiotics and Probiotics for Depression and Anxiety: A Systematic Review and Meta‐Analysis of Controlled Clinical Trials.” Neuroscience and Biobehavioral Reviews 102: 13–23. 31004628 10.1016/j.neubiorev.2019.03.023 PMC6584030

[brb371108-bib-0018] Marx, W. , A. Scholey , J. Firth , et al. 2020. “Prebiotics, Probiotics, Fermented Foods and Cognitive Outcomes: A Meta‐Analysis of Randomized Controlled Trials.” Neuroscience and Biobehavioral Reviews 118: 472–484. 32860802 10.1016/j.neubiorev.2020.07.036

[brb371108-bib-0019] Nikolova, V. L. , A. J. Cleare , A. H. Young , and J. M. Stone . 2023. “Acceptability, Tolerability, and Estimates of Putative Treatment Effects of Probiotics as Adjunctive Treatment in Patients with Depression: A Randomized Clinical Trial.” JAMA Psychiatry 80: 842–847. 37314797 10.1001/jamapsychiatry.2023.1817 PMC10267847

[brb371108-bib-0020] Page, M. J. , J. E. McKenzie , P. M. Bossuyt , et al. 2021. “The PRISMA 2020 Statement: An Updated Guideline for Reporting Systematic Reviews.” Bmj 372: n71. 33782057 10.1136/bmj.n71 PMC8005924

[brb371108-bib-0021] Price, R. B. , and R. Duman . 2020. “Neuroplasticity in Cognitive and Psychological Mechanisms of Depression: An Integrative Model.” Molecular Psychiatry 25: 530–543. 31801966 10.1038/s41380-019-0615-x PMC7047599

[brb371108-bib-0022] Roberts, A. L. , J. Liu , R. B. Lawn , et al. 2022. “Association of Posttraumatic Stress Disorder With Accelerated Cognitive Decline in Middle‐Aged Women.” JAMA Network Open 5: e2217698. 35771577 10.1001/jamanetworkopen.2022.17698 PMC9247738

[brb371108-bib-0023] Romo‐Araiza, A. , G. Gutiérrez‐Salmeán , E. J. Galván , et al. 2018. “Probiotics and Prebiotics as a Therapeutic Strategy to Improve Memory in a Model of Middle‐Aged Rats.” Frontiers in Aging Neuroscience 10: 416. 30618722 10.3389/fnagi.2018.00416 PMC6305305

[brb371108-bib-0024] Rudzki, L. , L. Ostrowska , D. Pawlak , et al. 2019. “Probiotic *Lactobacillus plantarum* 299v Decreases Kynurenine Concentration and Improves Cognitive Functions in Patients With Major Depression: A Double‐Blind, Randomized, Placebo Controlled Study.” Psychoneuroendocrinology 100: 213–222. 30388595 10.1016/j.psyneuen.2018.10.010

[brb371108-bib-0025] Salami, M. , and M. Soheili . 2022. “The Microbiota‐Gut‐ Hippocampus Axis.” Frontiers in Neuroscience 16: 1065995. 36620458 10.3389/fnins.2022.1065995 PMC9817109

[brb371108-bib-0026] Schneider, E. , J. P. K. Doll , N. Schweinfurth , et al. 2023. “Effect of Short‐Term, High‐Dose Probiotic Supplementation on Cognition, Related Brain Functions and BDNF in Patients With Depression: A Secondary Analysis of a Randomized Controlled Trial.” Journal of Psychiatry & Neuroscience 48: E23–E33. 36653035 10.1503/jpn.220117 PMC9854921

[brb371108-bib-0027] Sousa, G. M. J. , H. D. Q. Vargas , F. F. Barbosa , and N. L. Galvão‐Coelho . 2021. “Stress, Memory, and Implications for Major Depression.” Behavioural Brain Research 412: 113410. 34116119 10.1016/j.bbr.2021.113410

[brb371108-bib-0028] Tiller, J. W. 2013. “Depression and Anxiety.” Medical Journal of Australia 199: S28–S31. 25370281 10.5694/mja12.10628

[brb371108-bib-0029] Tsai, Y.‐C. , S. Wang , L.‐H. Cheng , O.‐J. Jeng , and F. Marotta . 2023. “Gerobiotics: Probiotics for Healthy Aging.” In Gut Microbiota in Aging and Chronic Diseases, edited by F. Marotta , 357–373. Springer International Publishing.

[brb371108-bib-0030] Xiang, S. , J.‐L. Ji , S. Li , et al. 2022. “Efficacy and Safety of Probiotics for the Treatment of Alzheimer's Disease, Mild Cognitive Impairment, and Parkinson's Disease: A Systematic Review and Meta‐Analysis.” Frontiers in Aging Neuroscience 14: 730036. 35185522 10.3389/fnagi.2022.730036 PMC8851038

[brb371108-bib-0031] Xu, F. , Q. Xie , W. Kuang , and Z. Dong . 2023. “Interactions Between Antidepressants and Intestinal Microbiota.” Neurotherapeutics 20: 359–371. 36881351 10.1007/s13311-023-01362-8 PMC10121977

[brb371108-bib-0032] Zareie, M. , K. Johnson‐Henry , J. Jury , et al. 2006. “Probiotics Prevent Bacterial Translocation and Improve Intestinal Barrier Function in Rats Following Chronic Psychological Stress.” Gut 55: 1553. 16638791 10.1136/gut.2005.080739 PMC1860130

[brb371108-bib-0033] Zhu, R. , Y. Fang , H. Li , et al. 2023. “Psychobiotic *Lactobacillus plantarum* JYLP‐326 Relieves Anxiety, Depression, and Insomnia Symptoms in Test Anxious College via Modulating the Gut Microbiota and Its Metabolism.” Frontiers in Immunology 14: 1158137. 37033942 10.3389/fimmu.2023.1158137 PMC10077425

