# Supplementary material for: Role of Probiotics in Management of Depressive Symptoms and Cognitive Impairment in Patients With Depression: An Updated Analysis of Trials
Source: Brain Behav. 2025 Nov 29;15(12):e71108. doi: 10.1002/brb3.71108 (PMC12665043; doi:10.1002/brb3.71108)
Supplement: Supplementary file 1 — Figure S1. Risk of bias graph. Figure S2. Funnel plot of effect of probiotics in improving cognitive functions in the selected participants of individual study. Figure S3. Funnel plot of effect of probiotics in reducing depressive signs and symptoms in the selected participants of individual study. [file BRB3-15-e71108-s001.docx]

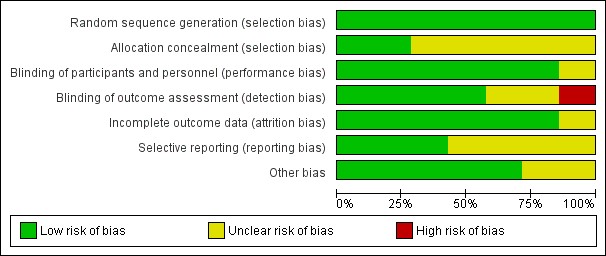


**Supplementary Figure 1**. Risk of bias graph.


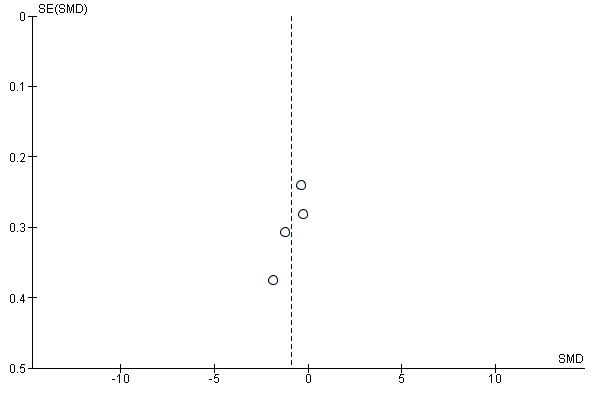


**Supplementary Figure 2**. Funnel plot of effect of probiotics in improving cognitive functions in the selected participants of individual study.


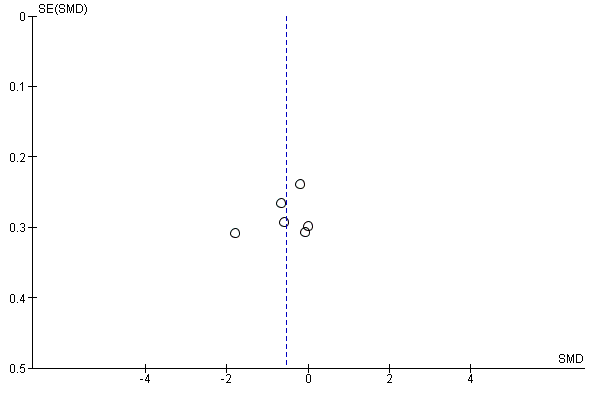


**Supplementary Figure 3**. Funnel plot of effect of probiotics in reducing depressive signs and symptoms in the selected participants of individual study.
