# Supplementary material for: Role of Probiotics in Management of Depressive Symptoms and Cognitive Impairment in Patients With Depression: An Updated Analysis of Trials
Source: Brain Behav. 2025 Nov 29;15(12):e71108. doi: 10.1002/brb3.71108 (PMC12665043; doi:10.1002/brb3.71108)
Supplement: Supplementary file 2 — Table S1. Search strategies of all databases. Table S2. Risk of bias assessment by Cochrane risk of bias tool. [file BRB3-15-e71108-s002.docx]

**Supplementary Table 1**. Search strategies of all databases.

| **Database** | **Search strategies** |
| --- | --- |
| PubMed | (Probiotic OR probiotics OR "Lactobacillus acidophilus" OR "Lactobacillus rhamnosus" OR "Bifidobacterium bifidum" OR "Bifidobacterium longum" OR "Lactobacillus helveticus" OR "Streptococcus thermophilus" OR "Bifidobacterium breve" OR "Bifidobacterium infantis" OR "Lactococcus lactis" OR "Lactobacillus plantarum") AND ("depression" OR "depressive disorder" OR "mood disorders" OR "cognition" OR "cognitive function" OR "neurocognition" OR "memory" OR "executive function") Filters: Randomized Controlled Trial |
| Google scholar | (Probiotic OR probiotics OR "Lactobacillus acidophilus" OR "Lactobacillus rhamnosus" OR "Bifidobacterium bifidum" OR "Bifidobacterium longum" OR "Lactobacillus helveticus" OR "Streptococcus thermophilus" OR "Bifidobacterium breve" OR "Bifidobacterium infantis" OR "Lactococcus lactis" OR "Lactobacillus plantarum") AND ("depression" OR "depressive disorder" OR "mood disorders" OR "cognition" OR "cognitive function" OR "neurocognition" OR "memory" OR "executive function") |
| Cochrane Central Register  of Controlled Trials | (Prob(probiotic OR probiotics OR “Lactobacillus acidophilus” OR “Lactobacillus rhamnosus” OR “Bifidobacterium bifidum” OR “Bifidobacterium longum” OR “Lactobacillus helveticus” OR “Streptococcus thermophilus” OR “Bifidobacterium breve” OR “Bifidobacterium infantis” OR “Lactococcus lactis” OR “Lactobacillus plantarum”) AND (“depression” OR “depressive disorder” OR “mood disorders”) AND (“cognition” OR “cognitive function” OR “neurocognition” OR “memory” OR “executive function”)iotic OR probiotics OR "Lactobacillus acidophilus" OR "Lactobacillus rhamnosus" OR "Bifidobacterium bifidum" OR "Bifidobacterium longum" OR "Lactobacillus helveticus" OR "Streptococcus thermophilus" OR "Bifidobacterium breve" OR "Bifidobacterium infantis" OR "Lactococcus lactis" OR "Lactobacillus plantarum") AND ("depression" OR "depressive disorder" OR "mood disorders" OR "cognition" OR "cognitive function" OR "neurocognition" OR "memory" OR "executive function") in Title Abstract Keyword - in Trials (Word variations have been searched) |

**Supplementary Table 2**. Risk of Bias Assessment by Cochrane Risk of Bias Tool.

|  | **Cochrane Risk-of-Bias Tool** | | |
| --- | --- | --- | --- |
|  | **Bias** | **Risk of bias** | **Author judgement** |
| Chahwan 2019 | Random sequence generation (selection bias) | Low Risk | Quote: The study mentions randomization, but it doesn't provide detailed information on the randomization process |
|  | Allocation concealment (selection bias) | Low Risk | Quote: Both researchers administering the trial as well as participants being unaware of group allocation throughout the trial and analysis of the results |
|  | Blinding of participants and personnel (performance bias) | Low Risk | Quote: ”The study was triple blinded which means participants were unaware of treatment they received |
|  | Blinding of outcome assessment (detection bias) | Unclear Risk | Quote: no data available |
|  | Incomplete outcome data (attrition bias) | Low Risk | Quote: reason for attrition was given and possible measure were conducted to meet the attrition |
|  | Selective reporting (reporting bias) | Unclear risk | No data available |
|  | Other bias | Unclear risk | No data available |
| Schneider 2023 | Random sequence generation (selection bias) | Low Risk | Quote: The study employed a conventional randomization software to allocate participants to the experimental or control group in a 1:1 ratio |
|  | Allocation concealment (selection bias) | Unclear Risk | Quote: “Insufficient detail to allow definitive judgement.” |
|  | Blinding of participants and personnel (performance bias) | Low Risk | Quote: Investigators and assessors were blinded during data collection and analysis, |
|  | Blinding of outcome assessment (detection bias) | Low risk | Quote: The text indicates that investigators and assessors were blinded during data collection and analysis |
|  | Incomplete outcome data (attrition bias) | Low Risk | Quote: Attrition was approached by modified intention-to-treat (ITT) approach, |
|  | Selective reporting (reporting bias) | Low Risk | Quote: Both significant and non-significant outcomes were reported |
|  | Other bias | Low Risk | Quote: “The study appears to be free of other sources of bias.” |
| Rudzkia 2018 | Random sequence generation (selection bias) | Low Risk | Quote: Participants were randomly assigned to either the placebo or pro biotic group using a computer-generated randomization list |
|  | Allocation concealment (selection bias) | Unclear risk | Quote: No data available |
|  | Blinding of participants and personnel (performance bias) | Low risk | Quote: The study was described as double-blinded, indicating that neither the participants nor the personnel involved in the study were aware of which treatment |
|  | Blinding of outcome assessment (detection bias) | Low Risk | Quote: The blinding of outcome assessment was adequately addressed in the study |
|  | Incomplete outcome data (attrition bias) | Low Risk | Quote: attrition did not significantly affect the study results |
|  | Selective reporting (reporting bias) | Low Risk | Quote: The primary and secondary outcome measures were outlined, |
|  | Other bias | Low Risk | Quote: “The study appears to be free of other sources of bias.” |
| Thuluva 2023 | Random sequence generation (selection bias) | Low risk | Quote: “study employed computer-generated blocked lists to randomize participants into groups, |
|  | Allocation concealment (selection bias) | Unclear Risk | Quote: “Insufficient detail provided for the judgement.” |
|  | Blinding of participants and personnel (performance bias) | Low Risk | Quote: The study was described as a double-blind trial, where both participants and personnel involved in administering the intervention and assessing outcomes were blinded to group assignments. |
|  | Blinding of outcome assessment (detection bias) | Unclear risk | Quote: did not mention blinding of outcome assessors |
|  | Incomplete outcome data (attrition bias) | Low Risk | Quote: although there was some participants who lost follow up but it does not affect the outcome |
|  | Selective reporting (reporting bias) | Unclear Risk | Quote: no data available regarding the selective reporting |
|  | Other bias | Unclear risk | Quote: no data available for other bias |
| Rita 2023 | Random sequence generation (selection bias) | Low Risk | Quote: The study employed random sequence generation to allocate participants to the pro biotic or placebo groups, which helps minimize selection bias. |
|  | Allocation concealment (selection bias) | Low Risk | Quote: The study used a double-blind design, where both participants and personnel involved in the study were unaware of group assignment |
|  | Blinding of participants and personnel (performance bias) | Low Risk | Quote: the study implemented blinding procedures to ensure that both participants and personnel involved in the study were unaware of group assignments, |
|  | Blinding of outcome assessment (detection bias) | Low Risk | Quote: The study implemented blinding procedures to ensure that outcome assessors remained unaware of participants' group assignments |
|  | Incomplete outcome data (attrition bias) | Low Risk | Quote: Reason for the drop out was mentioned |
|  | Selective reporting (reporting bias) | Unclear Risk | Quote: No data available |
|  | Other bias | Low risk | Quote: The study appears to be free of other sources of bias |
| Zhu 2023 | Random sequence generation (selection bias) | Low Risk | Quote: participants were randomly allocated |
|  | Allocation concealment (selection bias) | Unclear Risk | Quote: no specific data on allocation |
|  | Blinding of participants and personnel (performance bias) | Unclear Risk | Quote: no specific data available |
|  | Blinding of outcome assessment (detection bias) | High Risk | Quote: outcomes were assessed using standardized questionnaires (HAMA-14, HDRS-17, AIS-8) to evaluate anxiety, depression, and insomnia symptoms. Since these assessments rely on self-reporting by participant |
|  | Incomplete outcome data (attrition bias) | Unclear risk | Quote: no specific data available on attrition |
|  | Selective reporting (reporting bias) | Unclear Risk | Quote: no data available |
|  | Other bias | Low Risk | Quote: “The study appears to be free of other sources of bias.” |
| Nikolova 2023 | Random sequence generation (selection bias) | Low Risk | Quote: The study randomly assigned the particle according to ONSORT guidelines, that divide part |
|  | Allocation concealment (selection bias) | Unclear Risk | Quote: “Insufficient detail to allow definitive judgement.” |
|  | Blinding of participants and personnel (performance bias) | Low Risk | Quote: participants were blinded to their group assignment, and the success of masking was evaluated by asking participants to guess their allocation at the end of the study. |
|  | Blinding of outcome assessment (detection bias) | Low Risk | Quote: “The outcome assessors were also blinded to treatment allocation.” |
|  | Incomplete outcome data (attrition bias) | Low Risk | Quote: Although there were some attrition in study but they managed to reduce the risk by balancing the outcome by some methods |
|  | Selective reporting (reporting bias) | Low Risk | Quote: The study provided comprehensive reporting of outcomes, including primary and secondary outcome measures, as well as exploratory analyses. |
|  | Other bias | Low Risk | Quote: “The study appears to be free of other sources of bias.” |
